# Characteristics of Oligo-Recurrence and Treatment Selection in Non-Small Cell Lung Cancer

**DOI:** 10.3390/cancers17142293

**Published:** 2025-07-10

**Authors:** Dai Sonoda, Yasuto Kondo, Satoru Tamagawa, Masahito Naito, Masashi Mikubo, Kazu Shiomi, Kazuhiro Yasufuku, Yukitoshi Satoh

**Affiliations:** 1 Department of Thoracic Surgery, Kitasato University School of Medicine, 1-15-1 Kitasato, Minami-ku, Sagamihara 252-0374, Kanagawa, Japanshiomi@med.kitasato-u.ac.jp (K.S.); ysatoh@med.kitasato-u.ac.jp (Y.S.); 2 Division of Thoracic Surgery, Toronto General Hospital, University Health Network, Toronto, ON M5G 2C4, Canada; 3 Department of Thoracic Surgery, Kitasato University Medical Center, 6-100 Arai, Kitamoto 364-8501, Saitama, Japan; 4 Department of Surgery, University of Toronto, Toronto, ON M5S 1A1, Canada

**Keywords:** oligo-recurrence, local therapy, lung cancer, recurrence

## Abstract

Recent advances in technology and pharmacologic agents have significantly improved both local and systemic therapies, making the treatment of non-small cell lung cancer (NSCLC) more effective and less invasive. However, recurrence after radical resection remains a major clinical challenge. Among the various recurrence patterns, oligo-recurrence—particularly metachronous oligo-recurrence, characterized by a limited number of metastatic lesions appearing after a disease-free interval—has gained attention due to its potential for long-term survival and even cure through local therapy. Concurrently, systemic treatments have advanced with the development of molecularly targeted therapies and immune checkpoint inhibitors. Numerous studies have demonstrated their clinical efficacy, resulting in significant improvements in patient prognosis. Therefore, selecting an appropriate treatment strategy for recurrent NSCLC involves a broad spectrum of therapeutic options, including targeted therapies, immune checkpoint inhibitors, and conventional chemotherapy. Treatment decisions are particularly complex in cases of oligo-recurrence, where local therapy is feasible, making it challenging to choose the best approach from the available options. This narrative review summarizes current evidence from retrospective and ongoing prospective trials and discusses the clinical characteristics and treatment strategies for oligo-recurrent NSCLC.

## Simple Summary

Oligo-recurrence in non-small cell lung cancer (NSCLC) is generally defined as a limited number of metastatic recurrences following complete resection of the primary tumor. This condition is associated with a potentially favorable prognosis and, in some cases, the possibility of cure through local therapy. NSCLC is also a type of cancer for which systemic treatments, such as molecularly targeted therapies and immune checkpoint inhibitors, have recently been significantly developed. Local therapies, including surgical resection and radiation therapy, play an important role in the management of NSCLC, especially in cases of oligo-recurrence. Therefore, a clear understanding of the definition and clinical characteristics of oligo-recurrence is essential for selecting the most appropriate treatment strategy. A multicenter, randomized phase III clinical trial investigating treatment for oligo-recurrence in NSCLC is currently underway. Its results may guide future strategies for managing postoperative recurrence.

## 1. Introduction

Recent advances have significantly improved the treatment of lung cancer. However, recurrence remains common in non-small cell lung cancer (NSCLC) [1,2], and managing recurrence is one of the most significant challenges in NSCLC treatment. Generally, postoperative recurrence is considered a systemic disease, with chemotherapy being the standard treatment for recurrent NSCLC [3].

Hellman and Weichselbaum proposed a different concept, oligometastases, in 1995. They reported that, among patients with metastases, when the number of metastases is small, local therapy can be effective, potentially resulting in long-term survival in some patients [4]. Oligometastases is not a concept limited to specific cancers. Subsequent studies on oligometastases in lung cancer have revealed that local therapy can be effective, and this subset of patients may have a favorable prognosis [5,6,7,8].

The concept of focusing on the number of metastases is also reflected in the tumor node metastasis (TNM) classification of lung cancer. The 8th edition of the International Association for the Study of Lung Cancer TNM classification, published in 2017, divides distant metastases into M1b (a single distant metastasis to one organ other than the lungs) and M1c (multiple distant metastases to multiple organs other than the lungs). This classification is based on the clear prognostic difference observed between M1b and M1c [9,10].

Conversely, Niibe et al. proposed the concept of oligo-recurrence in 2006. This was based on the idea that the most important prognostic factor in oligometastases is the status of the primary tumor [11,12,13]. They defined oligo-recurrence as a small number of metachronous metastases treatable with local therapy while the primary tumor remains under control. Some studies have reported that oligo-recurrences exhibit different characteristics from oligometastases [14]. Understanding these differences may be helpful in managing lung cancer recurrence.

In this narrative review, we aimed to explore the background, definitions, treatment options, and future prospects of oligo-recurrence in lung cancer.

## 2. Materials and Methods

We searched the PubMed and Google Scholar databases using different combinations of search terms, including “local therapy”, “lung cancer”, “radiotherapy”, “surgery”, “metachronous”, “oligometastases”, and “oligo-recurrence”. Additionally, we reviewed prospective and retrospective studies, meta-analyses, systematic reviews, and other relevant publications. We also examined the reference lists of these articles, screening for further eligible studies.

Of the papers selected using these methods, we excluded those with incomplete data, studies that included cancers other than NSCLC, those that mixed oligo-recurrence and oligometastases, and papers written in languages other than English.

## 3. Characteristics of Oligo-Recurrence of NSCLC

### 3.1. Oligo-Recurrence and Oligometastases

Postoperative recurrence is generally considered a systemic disease [3]. This theory is based on the idea that recurrence and metastases reflect the dissemination of cancer cells throughout the body. However, in 1995, Hellman and Weichselbaum proposed the concept of oligometastases, which focuses on the number of metastatic lesions [4]. They suggested that patients with a limited number of metastases represent an intermediate state between localized and widely disseminated disease. Rather, it has been observed across various cancers, demonstrating that local therapy targeting oligometastatic lesions can lead to a favorable prognosis [15,16,17,18]. Several prospective trials in lung cancer have revealed that local therapy is effective for oligometastases, with a favorable prognosis for these patients [5,6,7,8].

In contrast, Niibe et al., recognizing that the primary tumor status is the most important prognostic factor in oligometastases, proposed the concept of oligo-recurrence in 2006. They defined it as a limited number of recurrences treatable with local therapy, provided the primary tumor remains under control [11,12,13]. Table 1 summarizes the characteristics of oligo-recurrence and oligometastases in patients with lung cancer. Oligo-recurrence typically refers to a limited number of recurrences following complete resection of the primary lung cancer.

Niibe et al. investigated the prognosis of patients with NSCLC who had brain-only oligometastases and were treated with stereotactic radiosurgery or stereotactic radiotherapy. When stratified by oligostatus, the 5-year overall survival (OS) rate was 0% in the synchronous oligometastasis group, compared to 18.6% in the oligo-recurrence group, demonstrating significantly better OS in the oligo-recurrence group [14]. In multivariate analysis, oligo-recurrence was the only significant independent factor associated with a favorable prognosis (hazard ratio: 0.253 [95% CI: 0.082–0.043], *p* = 0.025). This indicates that oligo-recurrence exhibits distinct characteristics when compared to synchronous oligometastases.

Similar to oligometastases, oligo-recurrence is not limited to specific types of cancer. The effectiveness of local therapy and the favorable prognosis associated with it have been reported across various cancer types in the context of oligo-recurrence [19,20,21].

Numerous studies have examined oligo-recurrence in patients with NSCLC. These studies consistently revealed that patients with oligo-recurrence generally have a more favorable prognosis and tend to benefit from local therapy [22,23,24,25,26,27,28,29,30,31,32,33,34,35,36,37,38].

Therefore, when recurrence is detected during postoperative surveillance, it is essential to assess whether the recurrence meets the criteria for oligo-recurrence. The characteristics of oligo-recurrence in NSCLC are diverse and will be discussed in detail below.

### 3.2. Characteristics and Epidemiology of Oligo-Recurrence of NSCLC

Table 2 summarizes studies focusing on oligo-recurrence following complete resection of NSCLC. Although some of these studies do not explicitly use the term “oligo-recurrence,” the inclusion criteria clearly align with its definition. Studies including patients with oligometastases and oligo-recurrences were excluded. The reported frequency of oligo-recurrence among patients with recurrence varies across studies. However, many studies reported that it accounts for approximately 30–50% of all recurrent cases [22,23,24,25,26,27,28,29,30,31,32,33,34,35,36,37,38]. While the definitions of oligo-recurrence and inclusion criteria differ among studies, oligo-recurrence represents a substantial proportion of patients with recurrent NSCLC.

Depending on cancer type, oligo-recurrence has been reported in approximately 40–50% of esophageal cancer [39,40], 40–70% of prostate cancer [41,42], 30% of breast cancer [43], and 50% of renal cell carcinoma [44]. In contrast, the incidence of oligo-recurrence in colorectal cancer is relatively low, at around 10% [45].

The prognosis of patients with oligo-recurrence is relatively favorable, with reported 5-year post-recurrence survival rates ranging from approximately 30% to 50%. Notably, favorable outcomes have been observed, particularly in patients who received local therapy. Additionally, among local treatment modalities, radiotherapy is the most frequently reported, followed by surgical resection. There have also been reports of photon radiotherapy, proton beam therapy [30], and microwave ablation [33], each demonstrating acceptable toxicity profiles and favorable prognoses.

Most studies on oligo-recurrence have been published since 2020, which may reflect the recent growing interest in this clinical entity.

### 3.3. Oligo-Recurrence and Post-Recurrence Cure

Niibe et al. highlighted the potential for achieving a cure after recurrence through radical local therapy for oligo-recurrence [13]. However, clinical reports on post-recurrence cures in lung cancer remain limited.

Sekihara et al. [46] examined patients who survived for 5 years after recurrence, categorizing them into two groups: the “cancer-controlled group” and the “cancer-bearing group.” The cancer-controlled group included patients who underwent local therapy for recurrence and had no detectable tumors on imaging, as well as those who received chemotherapy for recurrence and maintained either a complete or partial response without progression for 5 years. As a result, 3% of patients with recurrence fell into the cancer-controlled group. Of the 19 patients in this group, only two received chemotherapy alone as their initial treatment. The remaining 17 underwent radical local therapy such as surgery or radiotherapy. Notably, none of the patients in the cancer-controlled group died of lung cancer, although two died from other causes.

Sonoda et al. examined patients with postoperative recurrence of NSCLC who did not develop new lesions or experience regrowth of existing lesions until the end of follow-up [47]. Patients who had no new lesions or regrowth for 5 years after treatment for recurrence were considered cured. All of these patients received radical local therapy for their recurrence and had fewer than two recurrences. They experienced oligo-recurrence, suggesting that some cases may be curable.

These findings indicate that patients with lung cancer can be cured even after recurrence. They also highlight the critical role of local therapy in achieving this outcome. The potential for a cure offers significant hope for patients with recurrence, emphasizing the importance of selecting the appropriate treatment for oligo-recurrence.

### 3.4. Definition of Oligo-Recurrence in NSCLC

The concepts of oligometastasis and oligo-recurrence are crucial in lung cancer treatment. However, a significant challenge lies in the lack of standardized definitions. In clinical trials involving NSCLC, different definitions of oligometastasis have been applied across studies [48].

In response to this lack of consensus regarding the definition of oligometastatic diseases, including oligometastasis and oligo-recurrence, several academic societies have proposed classification frameworks. In 2019, the Lung Cancer Group of the European Organization for Research and Treatment of Cancer (EORTC) proposed a consensus definition for synchronous oligometastatic NSCLC [49]. This consensus was developed based on survey responses from thoracic oncology experts, including pulmonologists, medical oncologists, radiation oncologists, thoracic surgeons, and radiologists from various academic societies and geographical regions.

According to this consensus, synchronous oligometastatic NSCLC is defined as the presence of up to five metastases in no more than three organs, all of which are amenable to local therapy. However, discrepancies remain regarding the number and location of metastases considered acceptable by experts. Notably, while lung metastases were included as eligible sites, mediastinal lymph nodes were excluded. Additionally, the definition of synchronous oligometastatic disease was applied only to cases deemed curable with local therapy, excluding patients with metastases to the meninges, pericardium, pleura, mesentery, or bone marrow.

In 2020, the European Society for Radiotherapy and Oncology (ESTRO), in collaboration with the EORTC, proposed a classification system for oligometastatic diseases that considers both the timing and treatment context [50]. This system consists of nine clinical scenarios based on five key questions: whether the disease is induced, synchronous, or metachronous, and whether the lesions represent progression. Within this framework, oligo-recurrence is categorized as metachronous oligo-recurrence. According to this proposal, metachronous oligo-recurrence is defined as limited metastatic progression occurring more than 6 months after definitive local therapy for the primary tumor. It involves up to five metastases in no more than three organs, all amenable to local therapy.

Additionally, a joint ESTRO/American Society for Radiation Oncology (ASTRO) consensus in 2020 defined oligometastatic disease as the presence of 1–5 metastatic lesions, all of which can be safely treated with curative-intent local therapy [51].

Furthermore, in 2023, ASTRO and ESTRO published a clinical practice guideline on the management of oligometastatic NSCLC, in which they adopted the same definition of oligometastatic disease [52]. However, these classifications have not yet been widely adopted. Notably, the definitions vary, and no unified standards have been established. For instance, a recent systematic review reported that only 7.4% of studies on oligometastases fully adopted the ESTRO-EORTC classification [53]. In a survey of Korean radiation oncologists, only 43.3% reported applying the ESTRO-EORTC classification in clinical practice, citing complexity (66%) and lack of supporting evidence (30%) as primary reasons for non-adoption [54].

Kang et al. proposed a new classification system that stratifies oligometastases into three subgroups based on disease progression: synchronous, oligo-persistent, and oligoprogression/recurrence [55]. These different perspectives underscore the current lack of consensus on the definition of oligometastatic diseases.

Conversely, regarding oligo-recurrence, Niibe et al. defined it as the presence of 1–5 metastatic or recurrent cancerous lesions with a controlled primary tumor. These lesions are amenable to local therapies, such as surgery, radiation, or radiofrequency ablation, with or without systemic therapy [11,12,13].

However, studies investigating oligo-recurrence in lung cancer have often adopted different definitions for analysis. As summarized in Table 2, the most commonly used definition specifies a maximum of three recurrent lesions (eight studies), followed by five or fewer (four studies), and two or fewer (four studies). The scope of the included patients also varied, with some studies focusing solely on pulmonary recurrence, while others included only lymph node recurrence.

Recognizing the importance of long-term survival and potential curability in defining oligo-recurrence, Sonoda et al. stratified postoperative NSCLC recurrences by number and evaluated post-recurrence survival (PRS) [34]. They demonstrated that patients with two or fewer recurrent lesions had a favorable prognosis and potential for curative treatment. Moreover, they also proposed that a reasonable threshold for defining oligo-recurrence in NSCLC is one or two recurrences treatable with local therapy.

Although multiple definitions of oligo-recurrence exist and no unified consensus has been established, it is reasonable to exclude patients with six or more recurrent lesions from the oligo-recurrence category. Further studies are warranted to determine the appropriate cutoff number of recurrent lesions for defining oligo-recurrence in NSCLC.

### 3.5. Advances in Lung Cancer Treatment and Oligo-Recurrence

The primary local treatments for lung cancer are surgery and radiotherapy. Recently, thoracoscopic surgery has become more commonly used, allowing lung cancer surgeries to be performed with less invasiveness [56]. Thoracoscopic surgery is associated with better postoperative general health in many patients than conventional open thoracotomy [57]. Therefore, it also provides a variety of treatment options for patients after recurrence.

Thoracoscopic surgery reduces inflammatory reactions in the pleural cavity owing to a small skin incision. It minimizes adhesions and distortions of the mediastinal structures. Repeat thoracoscopic surgery can be easily performed in the ipsilateral thorax, even after a lobectomy [26]. Furthermore, with the advent of robot-assisted surgery, minimally invasive procedures may become even more feasible in the future [58,59].

Additionally, radiation therapy has become more effective in reducing toxicity and improving local control. Advances in computer technology have led to the development of intensity-modulated radiation therapy and image-guided radiotherapy. These techniques use imaging for highly precise radiation delivery. New techniques have also been developed to account for tumor movement caused by patient respiration [60,61,62,63]. Moreover, these advances allow for the delivery of higher doses to tumors while minimizing exposure to surrounding healthy tissues. Stereotactic body radiation therapy (SBRT), which delivers higher doses over a shorter period than conventional radiation therapy, is another useful treatment for lung cancer. Some reports suggest that the outcomes of SBRT for early-stage lung cancer are comparable to those of surgery [64,65,66]. Thus, local therapies have advanced significantly, offering potential benefits to more patients in the treatment of NSCLC recurrence.

Furthermore, significant progress has been made in systemic therapies in recent years. In addition to conventional chemotherapy, the development of molecularly targeted agents and immune checkpoint inhibitors has significantly improved systemic treatment options.

The discovery of epidermal growth factor receptor (EGFR) gene mutations in 2004 as a predictor of EGFR-tyrosine kinase inhibitor (TKIs) efficacy paved the way for identifying a series of driver gene mutations and translocations. These include anaplastic lymphoma kinase (ALK) rearrangements, c-ros oncogene 1 fusion gene, and rearranged during transfection proto-oncogene alterations. The identification of these driver gene mutations and translocations has been followed by the development of TKIs targeting each of them, demonstrating significant efficacy [67,68,69,70]. The introduction of these drugs has revolutionized the pharmacotherapy of lung cancer.

Patients with NSCLC harboring driver gene mutations or translocations have been treated with targeted kinase inhibitors according to their specific genetic abnormalities, leading to favorable prognoses. Furthermore, the recent approval of immune checkpoint inhibitors has further accelerated advancements in lung cancer treatment. These inhibitors, particularly for patients with positive programmed cell death ligand 1 expression, are expected to result in better outcomes for these individuals [71].

Improved systemic therapy has been reported to enhance the survival benefit of local therapy [72]. This indicates that local therapy for recurrence may become even more valuable in the treatment of recurrent NSCLC, especially as systemic therapies continue to advance.

Further studies are required to determine the optimal combination of drug and local therapies to achieve more effective treatment outcomes for oligo-recurrence in lung cancer.

### 3.6. Choice of Local Therapy Options in Oligo-Recurrence of NSCLC

Local therapies, such as surgical resection or radiation therapy, are commonly employed in patients with oligo-recurrent lung cancer. However, the appropriate local treatment modality remains a subject of debate.

While radiation therapy is frequently selected as the local treatment in studies of oligo-recurrence, surgical intervention has also demonstrated efficacy. For instance, Ishige et al. reported on seven patients with NSCLC who developed oligo-recurrence in the liver and subsequently underwent hepatectomy. Complete resection was achieved without complications in all cases [73]. The median survival time after hepatectomy was 24.0 months (range: 15.2–30.2 months). The authors concluded that hepatectomy may be as effective as multidisciplinary therapy for managing hepatic oligo-recurrence of NSCLC.

Sonoda et al. compared post-recurrence survival and post-recurrence progression-free survival (PFS) between patients with oligo-recurrence confined to the lungs who underwent radical local therapy. They divided the patients into surgery and radiation therapy groups. Regarding patient characteristics, those who underwent surgical resection were younger, and all had a performance status of 0, compared to those who received radiation therapy [31]. Notably, among patients treated with either resection or radiation therapy, the 5-year post-recurrence survival rates were 61.5% and 47.6%, respectively (*p* = 0.258). In comparison, the 5-year post-recurrence progression-free survival rates were 30.3% and 24.7%, respectively (*p* = 0.665). No significant differences were observed between the two treatment modalities. Based on these findings, the authors concluded that it is necessary to decide the method of local therapy individually, considering the general condition of the patient.

Currently, there is no definitive survival advantage associated with one local treatment modality over another. The selection of local treatment for recurrence should be based on a comprehensive evaluation of factors, such as the involved organ, number and location of recurrent lesions, patient’s general health status, personal preferences, and social background. Therefore, individualized therapeutic strategies are essential for optimizing outcomes in patients with oligo-recurrent disease.

### 3.7. Oligo-Recurrence and Gene Mutation

Molecularly targeted therapies have demonstrated efficacy in patients with lung cancer with gene mutations. Recently, their effectiveness has also been reported in patients with recurrent NSCLC. Moriya et al. evaluated the efficacy of EGFR-TKI as a first-line treatment for recurrent NSCLC harboring EGFR gene mutations [74]. They reported a median PFS and OS of 18 and 61 months, respectively, following EGFR-TKI therapy. In multivariable analysis, osimertinib exhibited a trend toward longer PFS (hazard ratio (HR) 0.41, 95% CI, 0.12–1.1; *p* = 0.071). Based on these findings, the authors concluded that EGFR-TKIs appear to be a reasonable first-line treatment option for selected patients with postoperative recurrent EGFR-mutated NSCLC.

Miyata et al. analyzed the long-term survival outcomes and prognostic factors of patients receiving EGFR-TKIs as first-line treatment for postoperative recurrent EGFR-mutated lung adenocarcinoma using a multi-institutional database [70]. They reported a median PFS and OS of 26.1 and 55.4 months, respectively. Based on these findings, the authors concluded that first-line EGFR-TKI treatment was generally associated with favorable survival outcomes in patients with postoperative recurrent *EGFR*-mutated lung adenocarcinoma.

Meanwhile, the phase III synchronous investigation of non-small cell lung cancer with the drug and ablative strategy trial investigated the role of first-line TKI therapy in patients with EGFR-mutated synchronous oligometastatic NSCLC (defined as ≤5 metastatic lesions). Patients were randomized to receive EGFR-TKI therapy alone or in combination with upfront radiotherapy. This study demonstrated that the combination group had significantly improved outcomes, with a median PFS of 12.5 months vs. 20.2 months (*p* < 0.001) and a median OS of 17.4 months vs. 25.5 months (*p* < 0.001), compared to the TKI-only group. These results indicate that the addition of upfront local therapy using radiotherapy significantly improves PFS and OS in patients with EGFR-mutated NSCLC [8].

In contrast, Sonoda et al. evaluated and compared the effects of EGFR-TKI therapy versus radical local therapy in patients with oligo-recurrent EGFR-mutated NSCLC [35]. The 5-year post-recurrence survival rates were 59.4 and 45.5% in patients who received radical local therapy and those who did not, respectively (*p* = 0.777). Multivariate analysis revealed that radical local therapies did not improve PRS in patients with oligo-recurrence (*p* = 0.551). Additionally, Tachibana et al. examined patients with EGFR mutations or ALK fusion genes who developed oligo-recurrence. They compared outcomes between those initially treated with local therapy and those who received molecularly targeted therapy. Their findings revealed no significant differences in post-recurrence OS (*p* = 0.324) and post-recurrence PFS (*p* = 0.426) [36].

Similarly, Morita et al. evaluated both post-recurrence and postoperative OS in patients with recurrent EGFR-mutated NSCLC. Their analysis demonstrated that the recurrence pattern—the presence or absence of oligo-recurrence—was not a significant prognostic factor (HR, 1.01; 95% CI, 0.67–1.54; *p* = 0.95) [75].

To date, there is no clear evidence supporting the efficacy of local therapy in patients with oligo-recurrence harboring EGFR mutations or ALK fusion genes. This may be attributed to the high efficacy of EGFR-TKIs and ALK-TKIs, which can lead to favorable prognoses even in the absence of local therapy.

However, Matsuguma et al. reported that although EGFR-TKI therapy in patients with EGFR mutation-positive tumors prolonged the median PFS, most patients experienced disease progression within 2 years [27]. Based on this, they suggested that definitive local therapy should be considered before initiating targeted therapies in patients with oligo-recurrences.

Additionally, local therapy may potentially cure recurrent lesions and delay molecularly targeted therapies [36,76]. Therefore, local therapy may be useful and should be considered even in patients with oligo-recurrence and gene mutations.

In addition, EGFR gene mutations have also been shown to be associated with disease progression in patients with NSCLC oligo-recurrence. Sonoda et al. investigated the clinical course of patients with oligo-recurrence who underwent curative local therapy. They found that the presence of EGFR gene mutations was significantly associated with disease progression after local therapy (odds ratio (OR), 3.90; 95% CI, 1.19–12.79; *p* = 0.025). This underscores the importance of close follow-up in patients with EGFR-mutated oligo-recurrence [38].

Accordingly, treatment strategies for these patients should be individualized, considering their general health status and social backgrounds.

### 3.8. Clinical Trials of Oligometastases and Oligo-Recurrence

Several randomized controlled trials have investigated the role of local therapy for oligometastases in NSCLC. Although the number of patients in these studies was limited, all have suggested that local treatment may lead to prolonged survival outcomes [5,6,7,8]. However, the only prospective study specifically focusing on oligo-recurrence is the JCOG2108 trial, conducted by the Japan Clinical Oncology Group (JCOG) [77]. This ongoing multicenter, randomized phase III trial targets patients with postoperative recurrence of completely resected NSCLC, characterized by three or fewer distant metastases and no EGFR or ALK gene mutations. Following initial treatment with immune checkpoint inhibitors, either alone or in combination with cytotoxic chemotherapy, patients were divided into maintenance therapy and local therapy groups. The primary endpoint of this study was OS. The results of this trial may serve as a critical reference for developing future treatment strategies for oligo-recurrence in NSCLC, and the outcomes of ongoing research are eagerly awaited. However, the JCOG2108 trial targeted patients without gene mutations, and the usefulness of local therapy in oligo-recurrence with gene mutations remains unclear. Therefore, prospective clinical trials targeting patients with oligo-recurrence and gene mutations are awaited.

### 3.9. Other Types of Oligo-Recurrence

In many cases of lung cancer, surgery is the primary curative treatment. Thus, oligo recurrence typically refers to a limited number of recurrences following surgical resection. However, Zhang et al. investigated the efficacy of salvage local therapy in patients who experienced oligo-recurrence after definitive chemoradiation [78]. They reported that salvage local therapy was significantly associated with improved OS (39.0 vs. 19.0 months, *p* = 0.01).

Similarly, Jin et al. examined patients who developed extracranial single-organ oligo-recurrence after receiving radical treatments for the primary lesion, such as complete resection, SBRT, or radical radiotherapy [79].

Their analysis demonstrated that both definitive local therapy (HR, 0.469; 95% CI, 0.262–0.837; *p* = 0.010) and targeted therapy with TKIs (HR, 0.442; 95% CI, 0.206–0.950; *p* = 0.036) were significantly associated with prolonged PFS.

Kim et al. investigated patients with NSCLC without oncogenic driver mutations who developed brain-only oligo-recurrence after receiving definitive treatment (such as surgical resection, concurrent chemoradiation, or radiotherapy) [80]. These patients underwent either local treatment or whole-brain radiation therapy, with a reported 5-year OS rate (from the date of treatment for brain metastases to the date of death from any cause) of 33.1%. The authors concluded that long-term survival could be achieved through local therapy.

These findings suggest that, even in patients who received nonsurgical primary treatment, local therapy may be an effective option for managing a limited number of recurrences. However, since surgical resection remains the standard initial treatment for lung cancer, patients who have undergone alternative therapies may have a poorer general health status. Therefore, careful consideration of the patient’s overall condition is essential when determining the appropriateness of local therapy in these cases.

### 3.10. Future Perspectives of Oligo-Recurrence of NSCLC

Recent advances in imaging techniques have enabled more accurate tumor detections [81,82]. Furthermore, the integration of artificial intelligence with imaging diagnostics has been reported to achieve high diagnostic performance [83,84].

Additionally, technological progress in liquid biopsy has facilitated the identification of tumor-derived somatic mutations in plasma through the detection of tumor DNA (tDNA), which aids in identifying tumor recurrence [85]. Furthermore, the presence of circulating tDNA in the peripheral blood allows for earlier detection of recurrence compared to conventional imaging methods [86]. Circulating tumor cells have also been reported to be useful for detecting recurrence [87,88].

As diagnostic technologies continue to evolve, enabling earlier detection of recurrence, it is expected that more patients with recurrence will be identified at the oligo-recurrence stage.

Furthermore, in oligometastatic NSCLC, patients in whom ctDNA was not detected prior to radiation therapy had longer PFS and OS, suggesting that they are more likely to benefit from radiation therapy. Therefore, ctDNA may be useful not only for detecting recurrence but also for predicting the efficacy of local therapy [89]. Consequently, determining whether recurrence constitutes an oligo-recurrence will become increasingly important in the management of recurrent lung cancer.

**Figure 1:**
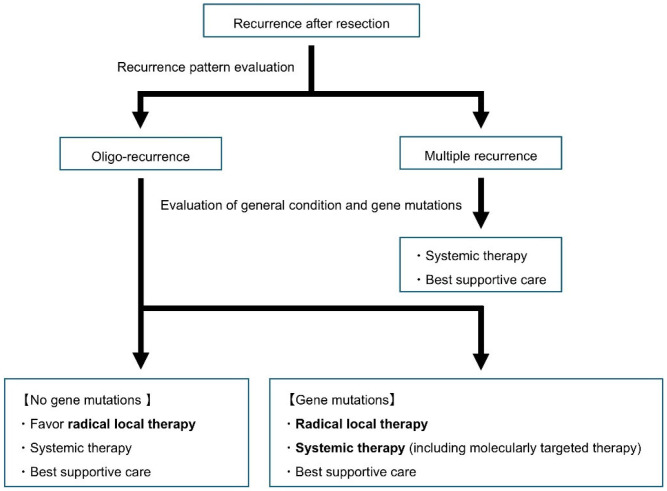
Treatment strategy for NSCLC oligo-recurrence.

The currently accepted treatment strategy for oligo-recurrence of NSCLC is illustrated in Figure 1. Upon identifying oligo-recurrence, it is essential to first evaluate the patient’s general condition and gene mutation status. For patients without such mutations and with good performance status, local therapy should be considered as an initial treatment option. Likewise, for patients with NSCLC harboring driver mutations amenable to targeted therapy, local treatment may also be appropriate if the patient’s condition is favorable.

However, several important questions remain unresolved, including whether systemic therapy should be combined with local treatment, the optimal timing and modality of local interventions, and how to best identify patients who are most likely to benefit from local therapy—potentially, through tools such as ctDNA. Further clarification of these issues requires multicenter collaborative studies and prospective clinical trials.

## 4. Conclusions

Oligo-recurrence in lung cancer represents a type of recurrence where curative local therapy can be effective, offering the potential for long-term survival or even a cure. With advances in both local and systemic therapies, the concept of oligo-recurrence is expected to play a progressively important role in clinical practice. Therefore, when recurrence is detected, it is essential to assess the number of recurrent lesions and whether they are amenable to local therapy. If recurrence meets the criteria for oligo-recurrence, careful consideration should be given to the patient’s general condition and preferences. Additionally, the feasibility of local therapy should be thoroughly evaluated.

## Figures and Tables

**Table 1:** Characteristics of oligo-recurrence and oligometastases in lung cancer.

	Oligometastases	Oligo-Recurrence
Reference	Hellman and Weichselbaum [4]	Niibe et al. [11]
Proposed year	1995	2006
Primary tumor	Uncontrolled or controlled	Controlled
Number of metastases/recurrences	Few	Few
Status of lung cancer	Stage IV or recurrent	Recurrent only

**Table 2:** Studies focusing on oligo-recurrence after complete resection of NSCLC.

Author, Publication Year	Research Period	Number of Recurrences	Number of Patients with Oligo-Recurrence	Target Patients	Treatment for Primary Lung Cancer	Treatment for Oligo-Recurrence	Frequency of Oligo-Recurrence	Prognosis of Patients with Oligo-Recurrence	Results
Yano et al., 2013 [22]	2007–2011	1–3	13	Distant metastases alone without primary site recurrence (excluding only brain metastases)	Complete resection	Local therapy, chemotherapy	33%	The median PFS was 20 months for patients who received local therapy	Local therapy is a viable first-line treatment option
Shimada et al., 2015 [23]	2000–2011	1–5	76	Distant recurrences in one or two sites	Complete resection	Local therapy, systemic treatment, no treatment	28%	N/A	It is important to combine local therapy with systemic therapy
Hishida et al., 2016 [24]	1993–2011	1–3	162	Loco-regional or distant recurrences within a single organ	Complete resection by lobectomy or greater resections	Definitive local therapy, chemotherapy, and best supportive care	21%	The 5-year PRS rate was 32.9% (definitive local therapy +: 38.6%, definitive local therapy −: 21.2%)	Initial definitive local therapy for oligo-recurrence achieved favorable PRS
Seol et al., 2017 [25]	2008–2013	1–5	31	Lymph node (the ipsilateral hilum, ipsilateral/contralateral mediastinum, and ipsilateral lower supraclavicular area) recurrence	Complete resection	Concurrent chemoradiotherapy, chemotherapy, and radiotherapy.	N/A	The 2-year OS rate was 58.4%	Salvage radiotherapy was effective with an acceptable level of toxicity
Han et al., 2020 [26]	2004–2014	1–5	102	Lung (both ipsilateral and contralateral) recurrence	Complete resection (lobar or sublobar resection for lung parenchyma and dissection of the mediastinal lymph nodes performed by VATS or open thoracotomy)	Operative and non-operative treatments (chemotherapy, radiotherapy, chemoradiotherapy, and best supportive care)	40%	The 5-year PRS rates in the operative and non-operative groups were 67% and 26%, respectively.	Operative treatment of pulmonary oligo-recurrence significantly prolonged the PRS.
Matsuguma et al., 2020 [27]	1986–2012	1–3	280	Distant and locoregional recurrences	Complete resection	Local therapy or systemic therapy, targeted therapy, chemotherapy	69%	N/A	Recent recurrence, oligo-recurrence, and definitive local therapy were associated with an improved median PR-PFS time and long-term PR-PFS rate in patients with recurrence
Yuan et al., 2020 [28]	2008–2015	1–3	119	Loco-regional confined to the lung lobe, hilar/mediastinal lymph nodes, bronchial stump, or chest wall.	Lobectomy and mediastinal lymph node dissection	Radical Local therapy, palliative local therapy, systemic chemotherapy, molecular targeted therapy, local palliative radiotherapy, and curative-intent supportive therapy	22%	The 5-year survival rate after recurrence was 10.8%	Patients with pulmonary solitary oligo-recurrence may achieve long-term survival.
Aoki et al., 2020 [29]	Patients treated for recurrence between 2011 and 2016	1–3	52	Nodular lesions in the thorax	Resection	Stereotactic body radiotherapy	N/A	The 3-year OS rate after radiotherapy was 67.8%	Post-operative salvage SBRT is a promising therapeutic option for patients with NSCLC and locoregional or intrathoracic oligo-recurrence.
Nakamura et al., 2020 [30]	2003–2016	N/A	33	Regional lymph node recurrence	Complete resection	Definitive salvage photon radiotherapy or proton beam therapy	N/A	The 3-year OS rate after radiotherapy was 63.8%	Salvage photon radiotherapy or proton beam therapy is an effective treatment for patients with NSCLC and oligo-recurrence in regional lymph nodes
Sonoda et al., 2021 [31]	1990–2008	1–3	97	Lung recurrences	Complete resection	Radical local therapy/chemotherapy/Best supportive care	N/A	The 5-year post-recurrence survival rates for patients who underwent resection and radiation were 61.5% and 47.6%, respectively.	No clear difference in prognosis was observed between patients who underwent resection or radiation.
Li et al., 2021 [32]	2010–2019	1–3	44	Thoracic recurrences (loco-regional lesions confined to lung lobe, hilar/mediastinal lymph nodes, bronchial stump, or chest wall)	Resection	Stereotactic body radiotherapy	N/A	The 5-year OS rate from the start of SBRT was 47.7%	SBRT is a promising salvage therapy with acceptable toxicity for postoperative thoracic oligo-recurrence in NSCLC.
Ni et al., 2021 [33]	2012–2020	1–5	103	Pulmonary recurrences	Radical R0 resection	All recurrent lesions underwent complete microwave ablation	N/A	The 5-year OS (calculated from the beginning date of pulmonary MWA to the date of death or the last follow-up) rate was 34.3%	Microwave ablation is an effective and safe treatment option for selected patients with pulmonary oligo-recurrence
Sonoda et al., 2022 [34]	1990–2009	1–2	214	Patients underwent local therapy for all recurrent lesions	Complete resection through lobectomy or a more extensive surgery with lymph node dissection	Complete surgical resection, gamma knife, stereotactic ablation radiotherapy, cerebral stereotactic radiosurgery, other radical radiation therapy of 45 Gy or higher doses, proton beam therapy, radiofrequency ablation, or cryotherapy.	37%	The 5-year PRS rate was 33.6%	A reasonable threshold to define oligo-recurrence in NSCLC is one or two recurrences that can be treated with local therapy
Sonoda et al., 2023 [35]	2004–2014	1–2	34	Patients with *EGFR*-mutated NSCLC	Complete resection through lobectomy or a more extensive surgery with lymph node dissection	Radical local therapy or systemic therapy	48%	The 5-year PRS rates in patients with *EGFR*-mutated NSCLC, who received radical local therapy for oligo-recurrence, and those who did not were 59.4 and 45.5%, respectively	Radical local therapy did not affect PRS in patients with oligo-recurrent *EGFR*-mutated NSCLC
Tachibana et al., 2024 [36]	2008–2020	1–3	66	Patients with lung adenocarcinoma with driver mutations	Surgical resection (lobectomy or more extensive pulmonary resection with mediastinal lymphadenectomy)	Local therapy or molecularly targeted therapy	N/A	N/A	Local therapies as a first-line treatment did not show significant differences in post-recurrence survival or PFS compared to molecular-targeted therapies.
Sonoda et al., 2025 [37]	2004–2015	1–2	125	Patients with oligo-recurrence	Complete resection through lobectomy or a more extensive surgery with lymph node dissection	Local therapy, systemic treatment, no treatment	46%	The 5-year PRS rates of patients who received radical local therapy and those who did not were 42.8% and 26.3%, respectively	The number of recurrences and receiving systemic therapy are important prognostic factors for patients with oligo-recurrence who undergo radical local therapy
Sonoda et al., 2025 [38]	2004–2016	1–2	88	Patients with oligo-recurrence who received local therapy	Complete resection through lobectomy or a more extensive surgery with lymph node dissection	Local therapy, systemic treatment, no treatment	46%	The 5-year PRS rate was 41.2%	EGFR positivity was associated with disease progression and lesion re-enlargement after radical local therapy.

N/A, Not Applicable; PFS, progression-free survival; PRS, post-recurrence survival; OS, overall survival; SBRT, stereotactic body radiotherapy; RT, radiotherapy; MWA, microwave ablation; VATS, video-assisted thoracoscopic surgery; NSCLC, non-small cell lung cancer; EGFR, epidermal growth factor receptor; R0 resection, complete surgical resection with negative margins.

## Data Availability

This article is a narrative review that synthesizes previously published studies. All data discussed are available from the cited sources in the reference list. No new data were generated or analyzed by the authors..

## Abbreviations

The following abbreviations are used in this manuscript:

ALK	Anaplastic Lymphoma Kinase
ASTRO	American Society for Radiation Oncology
EGFR-TKI	Epidermal Growth Factor Receptor Tyrosine Kinase Inhibitor
EORTC	European Organization for Research and Treatment of Cancer
NSCLC	Non-Small Cell Lung Cancer
OS	Overall Survival
PFS	Progression Free-survival
PRS	Post-recurrence Survival
SBRT	Stereotactic Body Radiation Therapy
TKI	Tyrosine Kinase Inhibitor
